# Do women’s autonomy lessen the brunt of unequal household responsibilities in the patriarchal context of semi-arid Northern Ghana?

**DOI:** 10.1371/journal.pone.0335142

**Published:** 2025-10-23

**Authors:** Sulemana Ansumah Saaka, Isaac Luginaah

**Affiliations:** Department of Geography and Environment, Faculty of Social Science, University of Western Ontario, London, Ontario, Canada; Bahir Dar University, ETHIOPIA

## Abstract

**Background:**

Gendered roles and household responsibilities characterize the social fabric of many patriarchal societies where traditional norms often dictate a clear division of labor between men and women. These deeply entrenched gendered expectations, for several decades, continue to shape the daily lives, opportunities, the physical and psychological wellbeing of women. In the semi-arid regions of Ghana, patriarchy and traditional gender norms are particularly pronounced.

**Methods and materials:**

Utilizing data from a cross-sectional survey (N = 1100 smallholder farmers), this study examined the role of women’s autonomy on shared-household responsibilities (SHRs). The composite measure of women’s autonomy was statistically robust (α = 0.823).

**Results:**

Women’s autonomy was associated with increased odds of shared responsibility for childcare (OR=1.872; P < 0.001) but less so with laundry (OR=0.635; P < 0.001) and food preparation (OR=0.764; P < 0.05). However, female-headed households were more associated with shared responsibility for laundry (OR=5.617; P < 0.001) and food preparation (OR=2.546; P < 0.05) with no significant observation for childcare. Regarding marital structure, polygamous households were less associated with shared responsibility for laundry (OR=0.233; P < 0.01) and food preparation (OR=0.361; P < 0.01) with no significant observation for childcare. Moreover, religion, age, and household wealth significantly predicted shared household responsibilities in the study context.

**Conclusions:**

Women’s autonomy is strongly associated with shared responsibility for childcare, but less associated with laundry and food preparation in Semi-Arid Northern Ghana, with notable differences based on household headship, marital structure, religion, age, and wealth. Findings underscore the need for more policies and programs that seeks to empower women at the household levels in rural agrarian settings.

## Introduction

Traditional gender roles and patriarchal norms have long dictated the structure and dynamics of household responsibilities in many societies across the globe. In many societies, men traditionally dominate the public sphere, while women are often associated with the private/domestic sphere [1,2]. Women worldwide spend long hours on unpaid domestic tasks (such as cooking, cleaning, and childcare). Despite being crucial for the functioning of family and society, this work is often unrecognized and undervalued [3,4]. More concerning is the fact that these discriminatory social norms and disproportionate burden of unpaid care work hampers most women’s ability to earn a living outside the domestic sphere, thereby rendering them financially dependent on their partners, and consequently increasing their vulnerability to poverty [3,5].

Although considerable progress has been made toward gender equality, studies however indicates that women continue to bear the brunt of house chores even when their earnings equal or surpasses their partners’ [6–10]. This ongoing gender disparity in housework implies that the gender revolution is either progressing slowly or have even stalled [11]. A major explanation for this observed trend, focuses on how gendered norms and expectations in patriarchal systems are ingrained from a very young age, influencing individuals’ identities and preferences, and how these norms manifest in house chores [12–14]. Scholarship on household labor, autonomy, and gender highlights how these persistent inequalities are shaped by socio-cultural norms and economic structures. Globally, it remains the stance of feminist economists that unpaid household labor, is largely feminized, undervalued and invisible in national policy fronts [15,16]. Consequently, the disproportionate burden of unpaid labor borne by women, reinforces existing traditional gender hierarchies, thereby limiting their autonomy and access to resources [17]. In regional contexts such as South Asia and Sub-Sahara Africa (SSA), patriarchal family structures intensify gendered expectations. For instance, studies in India indicate that even when women contribute financially, they rarely gain equal decision-making power within the household [18]. Likewise, in SSA, while female-headed households may demonstrate greater autonomy, they are constrained by economic precarity due to limited state support [19].

In Sub-Saharan Africa (SSA), women, particularly those confined to domestic spheres, have historically been relegated to subordinate roles, with limited autonomy in decision-making processes [20–23]. In Ghana, available evidence indicates that despite governmental and non-governmental interventions toward improvement of women’s autonomy through socio-economic empowerment [24], men continue to dominate household decision-making [25], which is a major antecedent to the unequal brunt of household chores that women continue to shoulder in patriarchal societies. In Semi-arid Northern Ghana in particular, patriarchy reinforces gender inequality by maintaining a social order where men largely hold power and predominate in roles of political leadership, moral authority, social privileges, and control over property including land for agriculture [26,27]. This system dictates that men are the breadwinners and decision-makers, while women are confined to domestic roles such as cooking, childcare, fetching of water and firewood among others. The persistence of these norms not only pose significant barriers to women’s empowerment but also obstructs equitable redistribution of household responsibilities. Consequently, the deeply entrenched gendered expectations in semi-arid regions of Ghana shapes both the daily lives and opportunities of women, as well as their overall wellbeing. For instance, available studies suggest that unequal burden of household responsibilities leads to less sleep time, increased depressive symptoms, reduced marital happiness, and overall poor physical and psychological wellbeing of women [28–31]. As a result, recent shifts towards women’s empowerment have begun to challenge entrenched gender norms and assigned roles, raising critical questions about the potential for such empowerment to foster more equitable distributions of household responsibilities [32].

Women’s empowerment which encompasses a broad range of social, economic, and political dimensions, in both the public and domestic sphere, is often measured by the ability of women to participate in and influence decisions that affect their own lives [33]. Other scholars have situated the definition of empowerment within contexts only where people’s ability to make strategic life choices was previously denied, and that the restoration of this ability to exercise choice can be thought of in terms of three interrelated dimensions: 1) *Resources*-that extend beyond material assets (i.e., *allocative resources)* to include human and social resources (acquired through social relationships within institutional domains such as the family and community) that enhance individuals’ capacity to make choices;2) *Agency*-ability to define one’s goals and act upon them, often operationalized as `decision-making’ and take several forms including bargaining/negotiation, deception/manipulation, subversion and resistance among others; and 3) *Achievements*- as a measure of desired well-being outcomes [34]. This definition draws a thin line between empowerment and autonomy-“the ability to define one’s own goals and act upon them without reference to notions of propriety and social standing” [34]. Thus, the current study conceptualizes women’s autonomy as *“Agency”* (i.e., women participation in decision-making that affects them), a condition for empowerment. Within the domestic sphere, women empowerment is therefore not barely a matter of individual agency but also a transformative process that can disrupt traditional power dynamics within families and communities and ensure that women’s voices are heard in decision-making processes, leading to policies, programs, and practices that address their needs and serve their interests. Empirical studies on the relationship between women’s empowerment and household responsibilities have produced mixed results. While some studies indicates that increased empowerment leads to a more equitable sharing of domestic tasks [35], others suggest that empowerment alone is insufficient to change deeply ingrained gender norms. For instance, studies show that in some cases, women’s increased participation in the workforce has resulted in a “double burden” where they continue to perform most household tasks in addition to their professional responsibilities [36].

Within the Ghanaian context, chieftaincy arrangement and inheritance system has also played a historic role in women’s autonomy. For instance, **in** matrilineal settings such as the Akan kinship arrangement in southern Ghana, traditional leadership is a dual gender system where every chief is paired with a queen mother, with the queen mother having the sole authority to nominate a qualified individual to assume the chieftaincy position when the stool becomes vacant [37]. Though such authorities in matrilineal systems undoubtedly elevates the social status and dignity of women, especially those from royal linages, paramount chiefs and sub-chiefs are largely well known and revered than queen mothers due to a colonial antecedent where queen mothers were ignored or disempowered by the British rule by relying heavily on paramount chiefs and sub-chiefs during indirect rule of the colonies [38]. Also, although globalization and gender equity movements have prompted the need for women empowerment and representation at all levels of leadership, queen mothers in Ghana nonetheless continue to face serious obstacles (e.g., limited ownership and/or access to resources) of precolonial female authority in a postcolonial society [38]. Like queen mothers in southern Ghana, qualification or the selection of women sub-chiefs in Northern Ghana (especially among Gonjas and Dagomba people) is based on the privilege of royal linage. Among the Dagbon for instance, female chiefs have historically been nominated based on royal lineage either as a daughter or granddaughter of the overlord, known as Ya Naa [39]. Wile these women sub-chiefs assume important traditional and political roles, particularly, during festivals and funerals of the Dagbon King, they remain constrained by several challenges including inadequate local recognition [39]. These traditional power imbalances translate into the persistent unequal decision-making autonomy confronting women not only at the local community and household levels, but also at the national level such as unequal representation at the Regional and National Houses of Chiefs.

Curated evidence further suggests that the marital structure, sociocultural understanding of bridewealth and marriage as an institution, all play a crucial role in women’s autonomy. For instance, Anfara et al. [40] in their exploration of the connection between household structure and women’s autonomy in Mauritania, unveiled that married women from polygamous households lacked decision-making ability regarding their own health, household purchases, as well as family visitations when compared to women from polygamous households. Also, ethnographic findings from Malenesia (i.e., regions in the South Pacific comprising Papua New Guinea, Vanuatu, New Caledonia, the Solomon Islands, West Papua and Fiji) suggest that although the payment of bridewealth may limits women’s autonomy, induce feelings of entitlement among husbands and in-laws, and possibly entrap women in toxic marital relationships in one breadth, it is however perceived as a collateral to women’s access to resources and guarantees improved social standing [41]. Likewise, payment of higher bridewealth is associated with increased women’s autonomy over their reproductive rights in Bangladesh [42]. However, within the patriarchal contexts of northern Ghana, Akurugu et al. uncovered that the payment of bride price, although normatively seen as a way of securing legitimacy for marriage to women, engenders women’s oppression [43] especially were the bride price is misogynistically interpreted as ownership of, and control over the bride [41]. Thus, while cultural practices such as bridewealth is a well revered marital arrangement in Northern Ghana, there is a strong call for the need to build on culturally appropriate notions of communitarianism through the philosophy of “Ubuntu” and the negotiation of women’s rights through indigenous systems such as the traditional courts.

Women decision-making autonomy is shown to hold much promise in fostering shared household responsibilities [35,44]. However, there is a dearth of studies evaluating this important topic in Ghana. The context of semi-arid Northern Ghana provides a rich setting for investigating these dynamics. The harsh environmental conditions and limited economic opportunities in Northern Ghana exacerbate the challenges faced by women, making it essential to understand the specific barriers to, and facilitators of shared household responsibilities. In the UWR of Ghana, where patriarchal norms are deeply ingrained, examining the link between women’s decision-making autonomy and shared household responsibilities will provide valuable insights into the broader implications of gender equity initiatives. Also, being the first to comprehensively explore this topic using a multifaceted approach that account for women participation in several measures of decision-making in the household setting, findings from this study will unearth novel insights in the study context with broader application across similar contexts in SSA. Based on the main research question (i.e., Do Women’s autonomy lessen the brunt of unequal household responsibilities in the patriarchal context of Semi-Arid Northern Ghana?), we hypothesize the following:

i) Women with greater autonomy will most likely enjoy shared household responsibilities than those who may lack autonomy
ii) Women from female-headed households will most likely benefit from shared household responsibilities than those from male-headed households
iii) Women in monogamous marital relationships will most likely benefit from shared household responsibilities than those in polygamous marital relationships.

### Theoretical framework

This research is grounded in the theoretical framework of gender and development (GAD), which emerged in the 1980s with focus on the socially constructed roles of women and men, and the impact of these roles on development processes [45]. GAD highlight the social construction of these relations as the basis of women’s oppression, questioning gender roles, or why women are largely assigned inferior roles [45,46]. Rooted in socialist feminism, GAD links production and reproduction relations, considering all aspects of women’s lives. Thus, the GAD approach advocates for transforming gender relations by addressing power imbalances and promoting gender equality [45]. GAD contrasts earlier women in development (WID) approaches that primarily focused on integrating women into existing development processes without challenging the underlying structures of inequality [47]. It does not focus exclusively on productive or reproductive aspects of women’s lives to the neglect of the other. Rather, it examines the nature of women’s contribution within the context of work done both inside and outside the household and rejects the public/private dichotomy that has been used as a mechanism to devalue family and household maintenance work performed by women [45]. Thus, in evaluating the role of women’s autonomy on shared household responsibilities in the patriarchal context of Semi-arid Northern Ghana, GAD serves as a fundamental theoretical framework for our analysis.

### Study context

The upper west region, located at the northwestern corner of Ghana, is bounded by Burkina Faso at the north and west, and lies between latitude 9.8°-11.0° North and longitude 1.6°-3.0° West. With a total land area of 18,476km2, the region forms 7.8% of the total national land area of Ghana [48]. It experiences a single maxima rainy season usually between May and October and characterized by a long dry season during which rainfed agriculture is practically impossible in the absence of irrigation [49]. The region has an average temperature of 28°C, peaking at about 38°C. In the past decades, temperatures in the region have increased by 1.7°C and projected to increase by 3°C by 2050 [49]. The declining trend in rainfall [50] and increasing temperatures in UWR signifies the vulnerability of smallholder farmers the region to climate change impacts including poor crop productivity, and food insecurity. Increasing temperatures, erratic rainfalls, and other climatic stressors (e.g., severe thunderstorms, droughts, and floods) in the region, presents challenges to smallholder farmers in the process of food production. Agriculture is the main livelihood activity in the region—employing slightly over 80% of the region’s population. All the three districts in study were ranked among the poorest in the country [51]. Characterized by harsh climatic conditions and limited resources, the UWR present unique challenges and opportunities for examining the issue of gendered household responsibilities. Traditionally, women in the communities of UWR disproportionately bear the brunt of household chores, including fetching water and firewood, cooking, cleaning, and caring for children and the elderly. These tasks are time-consuming and very laborious, leaving women with little time for selfcare, education, or income-generating activities. Moreover, women from UWR are shown to have lower levels of involvement in household decision-making in Ghana, relative to those from other regions [52].

## Materials and methods

### Data collection and sampling techniques

This study employed a cross-sectional design based on a broader survey on farmer livelihood and agricultural production (FLAP) in the UWR of Ghana administered in 2019 (July to August). The targeted population for this study was the primary farmer(s) of each framing household in the study communities. Thus, survey questionnaires were administered to the primary farmer of each farming household to respond on behalf of the household. The survey included questions on household demographics, agricultural production, household food security and post-harvest losses, household expenditure, livelihood activities, gender relations, as well as climate change adaptive capacity and resilience. The survey team consisted of three researchers and six local research assistants. Proficiency in local languages, familiarity with the study context (i.e., the Upper West Region), past research experience, as well as being a resident of the study community, were the criteria for the selection of the six research assistants. Each of the three researchers supervised two research assistants in each of the selected districts. Ethical approval for this research was granted by the University of Western Ontario Non-Medical Ethics Research Board. Thus, following the ethical guidelines of the University of Western Ontario’s Non-Medical Research Ethics Board, the research assistants were given five days intensive training on the survey instrument, ethics and safeguarding protocols. The research assistants signed an agreement of confidentiality to protect the privacy and anonymity of the study participants.

Prior to the data collection, and as part of the training, the survey questions were role played and extensively discussed to ensure the meaning of the questions was consistent across local languages and districts. Community leaders (i.e., opinion leaders) were also engaged by the research team to explain the purpose of the study. The research assistants sought oral consent from participants in their local languages. Only participants who consented to participate in the survey were asked further questions.

A multi-stage sampling technique was used to select 1100 smallholder farming households. First, three districts (Wa West, Lawra, and Nadowli-Kaleo) were selected using purposive sampling. The selection was based on the prevalence of impoverished smallholder farmers in the study districts [49]. This sampling technique was more convenient because it allows researchers to deal solely with the targeted population (in this case, smallholder farmers) [49]. At the District level, to help minimize the risk of biases in the selection process, a simple random sampling was used to select the study communities/villages in each of the three Districts. Finally, a systematic sampling (every fifth household selected to participate in the survey) was then used to select household units in the study communities, giving all farming households an equal chance of being included in the research survey. This study thus utilized data from a cross-sectional survey with smallholder farmers (n = 1100) as part of a Social Science and Humanities Research Council funded project. The sample was proportionately distributed among the three selected districts (i.e., Lawra = 295, Nadowli = 367, Wa West = 438) based on their populations [53].

### Measures

Outcome variables: The outcome variables for this study were derived from the following question: 1) Do you (*or your husband*) ever do the laundry; 2) Do you (*or your husband*) ever help with food preparation; and 3) do you (*or your husband*) ever help with childcare? (0 = No, 1 = Yes).

The main predictor variable (i.e., Women’s autonomy) for this study is a complex construct, from a series of questions on decision-making in the study households. A summative scale was created using Principal Component Analysis (PCA). The PCA was specifically employed to construct a single latent scale with eight (8) questions that enquired about who usually has the final say in household settings, including decision on: 1) health care, 2) large purchases such as furniture, Vehicle, etc., 3) small purchase for daily needs, 4) visits to family and relatives, 5) what food to eat each day, 6) what and where to plant, 7) sales of farm products, and 8) participation in local organizations (1 = Everyone contributes equally, 2 = Male Head/Father only, 3 = Female Head/Mother only, 4 Male relative, 5 = Female relative, 6=Both female and male). Women are considered to have decision making autonomy only in response categories 1 (Everyone contributes equally), 3 (Female Head/Mother only), 5 (Female relative), and 6 (Both female and male). This approach has been adopted from earlier studies [52,54,55], and anchored on Kabeer’s robust women’s empowerment model, particularly, “*Agency*”-participation in decision making process on matters that affect women [34]. Thus, the construct considered only response categories where women participated in decision-making. The Cronbach’s alpha for the scale was statistically robust (α = 0.823). To account for possible confounders, we controlled for sociodemographic factors such as the gender of respondent, gender of household head, marriage structure, age, religion, educational attainment, and household wealth. Data relevant to this study are available from the Western University Dataverse (Borealis) at https://doi.org/10.5683/SP3/JPD6ME. If further access of the data is needed, you may contact The Director, Office of Human Research Ethics, email: ethics@uwo.ca. Please quote project (REB)#: 114075, for further assistance.

### Data analysis

Descriptive statistics, univariate, and multivariable analyses have been conducted in this study. First, a descriptive statistics table has been used to provide an overview of the study population. Secondly, univariate analysis was conducted to understand the relationship between women’s autonomy and shared household responsibilities. Finally, given the binary nature of the outcome variables, logistic regression model was employed for multivariable analysis. A test of multicollinearity preceded the inclusion of variables in the multivariate analysis. Specifically, Variance Inflation Factor (VIF) command “vif” was applied to examine the presence of multicollinearity in all independent variables. All variables included in the multivariate analysis had VIF values ranging between 1–5 with an average VIF of 2.05, suggesting a low-to-moderate multicollinearity as established in peer-reviewed literature [56]. All statistical analyses were conducted in Stata version 18.

## Results and discussion

### Descriptive statistics

Table 1 present the descriptive statistics for participants’ sociodemographic information. Majority of the study participants were from male-headed households (93.18%), in monogynous marital relationships (82.45%), were Christians (61.45%), and had no formal education (67.18%). In terms of shared household responsibilities, except for childcare (65.73%), majority of the respondents indicated that male partners in the studied households do not provide support for laundry (77.73%) and food preparation (72.91%).

**Table 1:** Descriptive statistics of study variables.

VARIABL	FREQUENCY (%)
**Husband helps with childcare**	
Yes	723(65.73)
No	369(33.55)
**Husband helps with laundry**	
Yes	229(20.82)
No	855(77.73)
**Husband helps with food preparation**	
Yes	276(25.09)
No	802(72.91)
**Gender of household Head**	
Male	1025 (93.18)
Female	75 (6.82)
**Marriage structure**	
Monogamous	747 (82.45)
Polygamous	159 (17.55)
**Age**	
18–25	94 (8.55)
26–35	216 (19.64)
36–45	382 (34.73)
46–59	338 (30.73)
60+	70 (6.36)
**Religion**	
Christian	676 (61.45)
Muslim	186 (16.91)
Traditional/Other	238 (21.64)
**Education**	
Tertiary	47 (4.27)
Primary	130 (11.82)
Secondary	184 (16.73)
No formal	739 (67.18)
**Wealth**	
Richest	206 (18.73)
Richer	190 (17.27)
Middle	248 (22.55)
Poorer	238 (21.64)
Poorest	218 (19.82)

### A closer look at women’s decision-making autonomy

Table 2 present the percentage distributions of seven questions related to women’s decision-making autonomy in our study. Except for health (63.82%) and what food to eat daily (48.68%) where the a significant proportion of decision-making was done by “Female Head/Mother only” in the farming households, decision on large purchases (85.45%), purchase for daily needs (67.91%), visits to family and relatives (69.61%), participation in local organizations (75.34%), what and where to plant (72.82%), as well as decision on the sales of farm products (82.73%) were largely taken by the “Male Head/Father only” in the farming households, signifying male-centeredness on important households’ decision in the study context.

**Table 2:** Descriptive statistics of women’s decision-making autonomy in smallholder households.

Variables on household decision making	Responses in percentages					
	Everyone contributes equally	Male Head/Father only	Female Head/Mother only	Male relative	Female relative	Both female and male
Healthcare	5.82	10.91	63.82	9.73	6.91	2.73
Large purchases	6.73	85.45	5.00	0.45	2.27	0.09
Purchase for daily needs	6.82	67.91	7.36	0.45	0.09	17.36
Visits to family and relatives	8.55	69.61	4.46	0.45	0.09	16.83
Participation in local organizations	6.10	75.34	6.10	0.82	0.09	11.56
What food to eat daily	6.73	37.76	48.68	0.09	1.09	5.64
What and where to plant	6.64	72.82	5.91	0.82	0.45	13.36
Sales of farm product	6.45	82.73	5.18	0.64	0.09	4.91

### Univariate analysis of women decision-making autonomy and shared household responsibilities

Table 3 present the results for univariate analysis of women decision-making autonomy and shared household responsibilities. From the results, women’s autonomy was significantly more associated with shared responsibilities for childcare (OR=1.201; P < 0.001), but less with laundry (OR=0.733; P < 0.001), and food preparation (OR=0.743; P < 0.001) in the study context, a reflection of the gendered household responsibilities in Northern Ghana.

**Table 3:** Univariate Analysis of women decision-making autonomy and shared household responsibilities.

STUDY VARIABLS	LAUNDARY		FOOD PREPARATION		CHILDCARE	
	OR (SE)	CI (95%)	OR (SE)	CI (95%)	OR (SE)	CI (95%)
**Women’s autonomy**	0.733(0.062) ***	0.620 0.867	0.743(0.058) ***	0.637 0.867	1.201(0.080) **	1.053 1.370
*P < 0.05; **P < 0.01; ***P < 0.001,	Pseudo R2 = 0.0129; Number of observations = 1,084		Pseudo R2 = 0.0126; Number of observations = 1,078		Pseudo R2 = 0.0055; Number of observations = 1,092	

Coeff = Coefficient, SE = Standard Error, CI = Confidence Interval.

### Multivariable analyses of women decision-making autonomy and shared household responsibilities

Table 4 presents the results for women’s decision-making autonomy and shared household responsibilities. Consistent with the bivariate results, women’s decision-making autonomy was significantly more associated with shared responsibility for childcare (OR=1.872; P < 0.001) but less with shared responsibility for laundry (OR=0.635; P < 0.001) and food preparation (OR=0.764; P < 0.05). Compared to male-headed households, female-headed households were significantly more associated with shared responsibility for laundry (OR=5.617; P < 0.001) and food preparation (OR=2.546; P < 0.05) with no significant observation for childcare. Regarding marital structure, polygamous marriages were significantly less associate with shared responsibility for laundry (OR=0.233; P < 0.01) and food preparation (OR=0.361; P < 0.01) with no significant observation for childcare. Age was significantly associated with only childcare [46–59 years (OR=0.278; P < 0.01) and 60 + years (OR=0.227; P < 0.01)] (see Table 3). Moreover, religion was significantly associated with shared household responsibilities in the study context. For instance, relative to Christians, Muslims were significantly less associated with shared responsibility for laundry (OR=0.475; P < 0.01) and food preparation (OR=0.627; P < 0.05) but no significant observation for childcare. Likewise, traditional religious believers were significantly less associated with shared responsibility for laundry (OR=0.272; P < 0.001) and food preparation (OR=0.346; P < 0.001) but however, significantly more associated with shared childcare responsibility (OR=1.784; P < 0.01). Finally, household wealth became a significant predictor of shared responsibility for childcare but no significant observation for laundry and food preparation (see Table 4). That is, compared to the richest households, the middle-income (OR=1.655; P < 0.05), poorer (OR=2.649; P < 0.001), poorest households (OR=6.244; P < 0.001) were all significantly more associated with shared childcare responsibilities.

**Table 4:** Multivariable results of women decision-making autonomy and shared household responsibilities.

STUDY VARIABLS	LAUNDARY		FOOD PREPARATION		CHILDCARE	
	OR (SE)	CI (95%)	OR (SE)	CI (95%)	OR (SE)	CI (95%)
**Women’s autonomy**	0.635(0.076) ***	0.501 0.805	0.764(0.079) *	0.624 0.936	1.872(0.191) ***	1.531 2.287
**Gender of household Head (Ref: Male)**						
Female	5.617(2.749) ***	2.151 14.663	2.546(1.199) *	1.011 6.412	0.707(0.361)	0.259 1.927
**Marriage structure (Ref: Monogamous)**						
Polygamous	0.233(0.115) **	0.088 0.615	0.361(0.126) **	0.181 0.718	1.351(0.368)	0.791 2.305
**Age (Ref: 18–25 years)**						
26–35	0.720(0.328)	0.294 1.761	0.597(0.256)	0.257 1.386	0.512(0.234)	0.208 1.256
36–45	0.452(0.204)	0.187 1.095	0.509(0.212)	0.224 1.155	0.494(0.221)	0.205 1.190
46–59	0.417(0.190)	0.170 1.018	0.454(0.192)	0.198 1.040	0.278(0.125) **	0.114 0.673
60+	0.480(0.278)	0.154 1.495	0.547(0.286)	0.196 1.528	0.227(0.122) **	0.079 0.653
**Religion (Ref: Christian)**						
Muslim	0.475(0.133) **	0.274 0.823	0.627(0.149) *	0.393 1.001	1.478(0.332)	0.951 2.298
Traditional/Other	0.272(0.090) ***	0.142 0.522	0.346(0.094) ***	0.203 0.591	1.784(0.419) **	1.125 2.830
**Education (Ref: Higher)**						
Secondary	1.520(0.735)	0.589 3.923	1.508(0.724)	0.588 3.866	0.540(0.253)	0.215 1.353
Primary	0.635(0.294)	0.256 1.574	1.016(0.458)	0.419 2.461	0.481(0.205)	0.208 1.113
No formal education	0.756(0.330)	0.321 1.778	1.138(0.489)	0.490 2.643	1.261(0.516)	0.565 2.814
**Wealth (Ref: Richest)**						
Richer	0.846(0.260)	0.463 1.548	0.795(0.224)	0.456 1.383	1.586(0.402)	0.964 2.608
Middle	0.898(0.254)	0.515 1.564	0.960(0.246)	0.580 1.589	1.655(0.393) *	1.038 2.638
Poorer	1.040(0.296)	0.595 1.819	0.981(0.254)	0.590 1.632	2.649(0.649) ***	1.639 4.283
Poorest	1.007(0.324)	0.535 1.894	0.929(0.273)	0.522 1.653	6.244(1.909) ***	3.429 11.369
**Pseudo R2**	0.1210		0.0710		0.1656	
**Number of observations**	896		893		905	

*P < 0.05; **P < 0.01; ***P < 0.001, Coeff = Coefficient, SE = Standard Error, CI = Confidence Interval.

## Discussion

Guided by the theoretical framework of gender and development (GAD), in the patriarchal context of semi-arid Northern Ghana, this study found that women’s autonomy is strongly associated with shared responsibility for childcare with no significant observation for laundry and food preparation. However, female-headed households were more likely to be associated with shared responsibility for both laundry and food preparation. These findings can be understood in the context of ethno-cultural and social dynamics prevalent in this region where childcare is often perceived as a communal responsibility while laundry making, and food preparation are perceived as feminine roles. Consistent with earlier studies [57,58], in most parts of Africa including Northern Ghana, child caregiving has historically been a communal effort aimed at promoting the child’s physical growth, reproductive development, and skills acquisition. The high value placed on children and their upbringing within Northern Ghanaian society means that childcare responsibilities are more likely to be shared by both male and female family members. Conversely, laundry and food preparation are traditionally viewed as women’s tasks in the patriarchal context of Northern Ghana [59,60]. These activities are deeply embedded in cultural norms that dictate specific domestic roles for women. Despite gains in women’s autonomy, women still find it challenging to shift these entrenched gender roles. Most men in patriarchal contexts tend to resist participating in these tasks due to societal expectations, and for the fear of stigmatization. For instance, a recent study from Ghana noted that even though men in both urban and rural areas of the country are willing partake in house chores, especially, during their partner’s pregnancy, they however misogynistically perceived it as a “potentially dangerous” disruption to the existing gendered division of labor, capable of stimulating laziness among their female partners [61]. Thus, even though much progress has been made in gender relations in Ghana, the prevalence of outdated cultural norms and beliefs in typical tradition Ghanaian communities poses a major challenge to achieving gender equity both in the private and public sphere. Furthermore, polygamous marriages were less associated with shared responsibilities for laundry and food preparation, a finding that can be attributed to the distinct domestic dynamics within polygamous households, which typically involve multiple wives with each managing their own set of household tasks. Also, the presence of multiple wives allows for a division of labor that reinforces traditional gender roles. Moreover, in polygamous marriages, the household structure can lead to competition among wives [62], further entrenching traditional roles and reducing men’s cooperation in domestic tasks. This finding underscores how the polygamous marital structure may perpetuate or even intensify traditional gender norms in the study context, leading to a clear demarcation of domestic responsibilities that limits the sharing of household chores between men and women. With regards to religious affiliations and shared household responsibilities, both Muslim and traditional religious believers exhibited lower odds of shared responsibility for laundry and food preparation in the study context, relative to Christians. Studies show that although Islamic laws inherently endorses gender equality, the interplay of cultural norms and localized traditions often influence its practical execution, leading to skewed gendered household roles [63]. Thus, the acculturation of Islam with patriarchal norms in the study context is a possible reason for the observed similarity between Islamic and traditional religious men’s lower likelihood of practicing shared responsibility for laundry and food preparation with their partners. Nevertheless, traditional religious believers showed higher odds of shared childcare responsibility relative Christians, indicating a divergence from other domestic tasks. This finding reflects cultural values that emphasize collective childcare practices within communal settings of Northern Ghana [57]. Overall, these findings highlight the influence of religious beliefs and cultural norms on the division of domestic labor in semi-arid Northern Ghana. Thus, while there are variations across different religious groups, the study underscores the persistence of traditional gender roles in shaping domestic responsibilities, particularly in tasks like laundry and food preparation.

With regards to household headship, men from female-headed households were more associated with shared responsibility for laundry and food preparation than those from male-headed households. Female-headed households, which often arise due to circumstances such as widowhood, divorce, or male migration in the patriarchal contexts [25], face unique challenges that necessitate a departure from traditional gender roles. Women, as heads of such households, must delegate responsibilities to other household members, including older male children and extended family members to ensure that chores like laundry and food preparation are adequately managed. In contrast, male-headed households typically adhere more strictly to traditional gender norms, where domestic chores are predominantly considered women’s duties [59,60]. Additionally, age significantly predicted shared responsibility for childcare in the study context. Specifically, individuals aged 46–59 years as well as those aged 60 years and above, exhibited lower odds of engaging in childcare responsibilities, a trend that may reflect generational differences in attitudes towards childcare within semi-arid Northern Ghana. Older individuals, particularly those aged 46 years and above, may adhere more strongly to traditional gender roles where childcare is predominantly seen as the responsibility of younger adults or women. They may prioritize other roles within the household or community, such as providing economic support or leadership, while leaving childcare duties to younger generations. Furthermore, older individuals in these age brackets may face physical limitations that restrict their ability to actively participate in childcare activities. These findings feature the interplay between age, cultural norms, and physical capabilities in shaping the division of childcare responsibilities within households in semi-arid Northern Ghana. Understanding these dynamics is crucial for developing targeted interventions to promote more equitable distribution of childcare duties across different age cohorts. Lastly, a significant association between household wealth and shared domestic tasks was observed. Specifically, compared to the richest households, men from middle-income households had higher odds of engaging in shared childcare responsibilities. Similarly, men from the poorer households show even higher odds, and the poorest households exhibit the highest odds of participating in shared responsibilities. These results suggest that household wealth plays a crucial role in determining the extent to which domestic responsibilities are shared. In wealthier households, there may be greater access to resources and services that alleviate the need for shared responsibilities, such as hiring domestic help or purchasing time-saving devices. Conversely, in middle-income, poorer, and poorest households, economic constraints may necessitate a more collaborative approach to managing household tasks among family members. These findings collaborate with that of earlier studies by Singh & Pattanaik [64] who observed that women belonging to households of lower socio-economic status, engages more in unpaid domestic activities. Besides, socio-economic status can influence cultural norms and expectations regarding gender roles and division of labor within households. Overall, these findings accentuate the complex interplay between household wealth, socio-economic factors, and cultural norms in shaping patterns of shared responsibilities within households.

### Study limitations

The findings of this study should be taken in the light of some noteworthy limitations. First, the quantitative and cross-section nature of the study implies that interpretation of the findings is limited to statistical association, not causal effects. Future studies can benefit from a mixed method approach. Also, the tendency of social desirability can lead to biased responses, thereby influencing the findings. Last, but not least, the binary nature of the outcome variables (i.e., “yes” or “no” responses to responsibilities sharing) may be a simplification of a complex reality as this approach do not capture the degree or frequency of responsibility sharing. For example, responding “yes” to sharing childcare responsibility could range from occasionally helping for a few minutes to equally sharing daily childcare tasks. This limitation could obscure subtle but important effects and may explain some of the weaker associations observed in the study. Thus, we call for future studies to consider exploring the frequency of responsibility sharing, which constitute a crucial dimensions of household responsibilities. That notwithstanding, this study has made a significant contribution to the existing literature on gender equity in the division of household labor, with valuable insights for women empowerment in the study context and similar contexts in Africa.

## Conclusion and recommendations

This study highlights that women’s autonomy is more associated with shared childcare responsibilities but not with laundry and food preparation in the study context with noteworthy differences based on household headship, marital structure, religion, age, and household wealth. The findings are thus reflective of the widespread prevalence of entrenched traditional gender norms and feminizations of household responsibilities in semi-arid Northern Ghana and similar contexts across SSA that continue to limit women’s ability to actively participate in decision-making processes that directly affects them both within and outside of the domestic sphere. Based on the findings, several policy recommendations are apparent including the need for the promotion of gender equality at the household level. This is achievable through the implementation of educational programs and public campaigns that encourage men to partake in domestic responsibilities (including laundry making and food preparation) within the study context and across similar context elsewhere in the SSA. This can be feasible through policies that promote paid paternity leaves, or dual parental leaves with tax incentives. Policies that reward co-parenting have the potential to shift household dynamics and encourage more equitable gender roles. Also, there is a pressing need to identify and provide social and economic support to female-headed households, particularly, the poorer and poorest households. Existing social security programmes such as the Livelihood Empowerment Against Poverty (LEAP) introduced by the Government of Ghana (GOG) in 2008, could be broadened to include equitable home responsibilities as one of the desired goals. This will not only translate into overall physical and psychological wellbeing of impoverished women in patriarchal systems but will concurrently offer them the necessary opportunity to partake in economic ventures outside the domestic sphere. Moreover, cultural and religious leaders must be engaged to dismantle the entrenched patriarchy and gendered household roles embedded within these very cultural and religious contexts. Engaging cultural and religious leaders is crucial for reshaping norms in patriarchal societies as their influence can legitimize gender-equitable sensitization programs, helping dismantle deeply rooted beliefs about household roles. Policy interventions should therefore include partnerships with these leaders to promote shared responsibilities and women’s autonomy as culturally and religiously acceptable, ensuring broader community acceptance and lasting social change in the study context and similar contexts across SSA.
